# Preservative Contact Hypersensitivity among Adult Atopic Dermatitis Patients

**DOI:** 10.3390/life12050715

**Published:** 2022-05-11

**Authors:** Dominik Németh, Erzsébet Temesvári, Péter Holló, Györgyi Pónyai

**Affiliations:** 1 Department of Dermatology, Venereology and Dermatooncology, Semmelweis University, 41 Mária Street, 1085 Budapest, Hungary; temesvari.erzsebet@med.semmelweis-univ.hu (E.T.); hollo.peter@med.semmelweis-univ.hu (P.H.); ponyai.gyorgyi@med.semmelweis-univ.hu (G.P.); 2 Károly Rácz Doctoral School of Clinical Medicine, Semmelweis University, 26 Üllői Street, 1085 Budapest, Hungary

**Keywords:** adult atopic dermatitis, contact allergy, preservatives, patch test

## Abstract

Atopic dermatitis (AD) is a chronic inflammatory disease characterised by an impaired skin barrier. The prolonged use of topical preparations containing medications, emollients, fragrances and preservatives may increase the risk of contact hypersensitivity (CHS). In the Allergy Outpatient Unit of the Department of Dermatology, Venereology and Dermatooncology of Semmelweis University, 5790 adult patients were patch tested between 2007–2021 with the European Environmental Baseline Series according to international standards. Among all the tested adult patients, 723 had preservative CHS (PCHS) and 639 had AD. Among the 723 PCHS patients, 68 (9.4%) had AD; the female to male ratio was 3:1 in this group. Out of 639 AD patients, 68 had PCHS (10.6%). In the AD-PCHS group, 83.8% had CHS to methylisothiazolinone (MI) (tested from 2014), 36.8% to Kathon CG^®^, 16.2% to methyldibromo-glutaronitrile, 11.8% to paraben, 7.4% to formaldehyde, 4.4% to para-tert-butylphenol-formaldehyde resin and 1.5% to Quaternium-15. The most common concomitant PCHS combination was Kathon CG^®^ + MI. Most patients (32.4%) belonged to the age group of 21–30, and skin symptoms affected mostly the limbs and face. The most common other concomitant allergens were nickel, lanolin alcohol and balsam of Peru. Preservatives (especially MI and Kathon CG^®^) are important contact allergens in adult AD, mostly among young women. The rate of AD in the PCHS group and the rate of PCHS in the AD group is remarkable; thus, the role of PCHS should be highlighted in the topical therapy and in the prevention of possible AD exacerbations.

## 1. Introduction

Atopic dermatitis (AD) is a common, relapsing, chronic inflammatory skin disease with scaly, pruritic, erythematous skin symptoms. It is characterised by skin barrier impairment in both lesional and non-lesional skin regions [1,2,3,4].

Patients with AD are treated locally, mostly with emollients, moisturisers, topical corticosteroids or calcineurin inhibitors. Several factors can modify the effectiveness of the therapy: stress, infections, lack of compliance and contact allergen exposures may trigger exacerbations. Because of the damaged skin barrier (higher chance of allergen penetration) and the long-term local therapy, there can be an increased risk of developing a contact hypersensitivity in AD patients [1,2,3,4,5,6,7,8].

Plenty of cosmetical and dermatological products contain not only ingredients which are helpful in care and treating AD, but fragrances and preservatives as well. An observation of a higher risk of CHS to preservatives among AD patients has been published, but the number of publications regarding preservative CHS and adult AD is very limited [2,8,9,10,11].

## 2. Materials and Methods

During our examination (2007–2021), 5790 consecutive adult (≥18 years) patients were patch tested with the European Environmental Baseline Series (EEBS). Out of them, 723 adult patients had PCHS and 639 had AD. Among them, data of adult PCHS AD patients (*n* = 68) were analysed.

Patch testing took place in the Allergy Outpatient Unit and Laboratory of the Department of Dermatology, Venereology and Dermatooncology of Semmelweis University between 2007–2021 on adult AD patients. The EEBS produced by Brial allergEAZE GmbH (Greven, Germany) were used (Section 3.5). Most of the antigens were dissolved in Vaseline. Formaldehyde, propylenglycol, Kathon CG^®^ (methylchloroisothiazolinone/methylisothiazolinone [MCI/M] 3:1) and methylisothiazolinone (MI) were used in the aqueous phase. Allergens of the EEBS are regularly revised and new ones can be included in the series [12,13,14,15,16,17,18,19]. The exact period of testing with the allergens and their patch testing concentrations are also listed in Section 3.5. The allergens were fixed on the patients’ asymptomatic back skin by a Curatest plaster (Lohmann & Rauscher International GmbH & Co., KG D-56579 Rengsdorf, Germany). The tests were performed according to international standards in a 48-h occlusion. Skin reactions were evaluated in 20–60 min, on day 2 (D2), D3, D4, and on D7. Written informed consent was granted from all patients before performing patch tests. Pregnant and breast-feeding women were not tested.

Data of adult AD patients who had positive patch test reaction to at least one of the following seven EEBS preservative allergens were evaluated: paraben, Kathon CG^®^, MI, formaldehyde, Quaternium-15, para-tert-butylphenol formaldehyde resin (PTBP-formaldehyde resin), methyldibromo-glutaronitrile (MDBGN).

## 3. Results

### 3.1. AD Patient Population

Out of the total tested adult patient population (*n* = 5790), 639 patients (11.03%) had AD.

There were 390 (61,03%) adult AD patients who had at least one CHS in the EEBS, and 68 (17.4%) had at least one positivity to preservatives.

**Figure 1:**
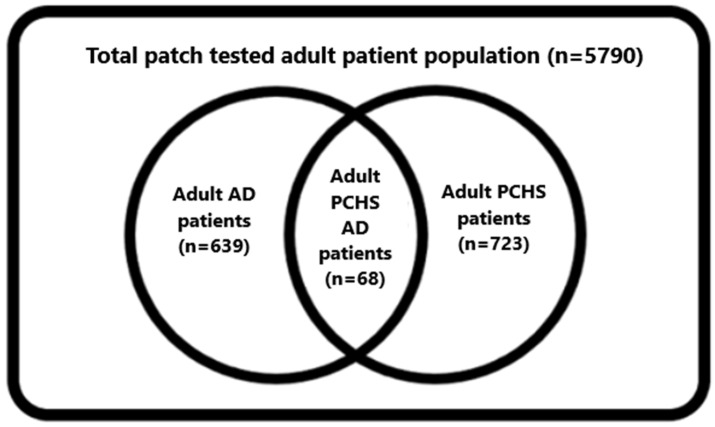
Distribution of the patch-tested patient population.

Among the tested 639 adult AD patients, 68 had at least one positivity to preservatives (10.6%) (Figure 1).

### 3.2. PCHS Patient Population

From the 5790 tested patients, 723 (12.5%) had CHS to at least one preservative. Out of them, 68 had AD (9.4% of the preservative CHS population) (Figure 1).

### 3.3. Adult AD Population with PCHS (n = 68)

1. Gender distribution:

In this study, 75.0% of patients were female and 25.0% were male. The female to male ratio was 3:1.

**Figure 2:**
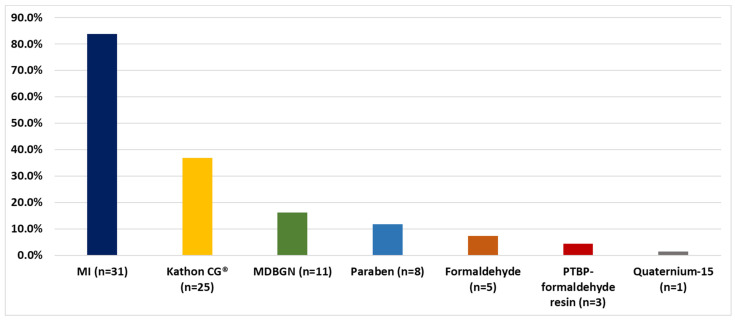
PCHS adult AD patients according to allergens. MI tested from 2014 (*n* = 37).

2. PCHS and polysensitivity:

Out of the 68 patients, 83.8% were MI (tested from 2014, *n* = 37), 36.8% Kathon CG^®^, 16.2% MDBGN, 11.8% paraben, 7.4% formaldehyde, 4.4% PTBP-formaldehyde resin and 1.5% Quaternium-15 hypersensitive (Figure 2).

**Figure 3:**
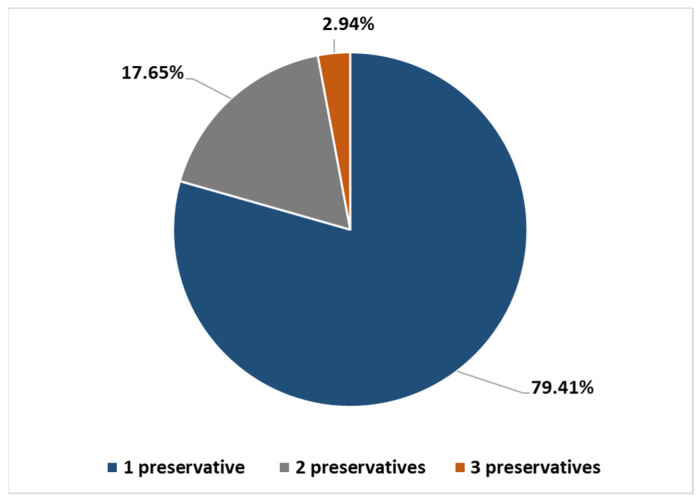
Proportion of PCHS and polysensitivity in the adult AD group.

**Figure 4:**
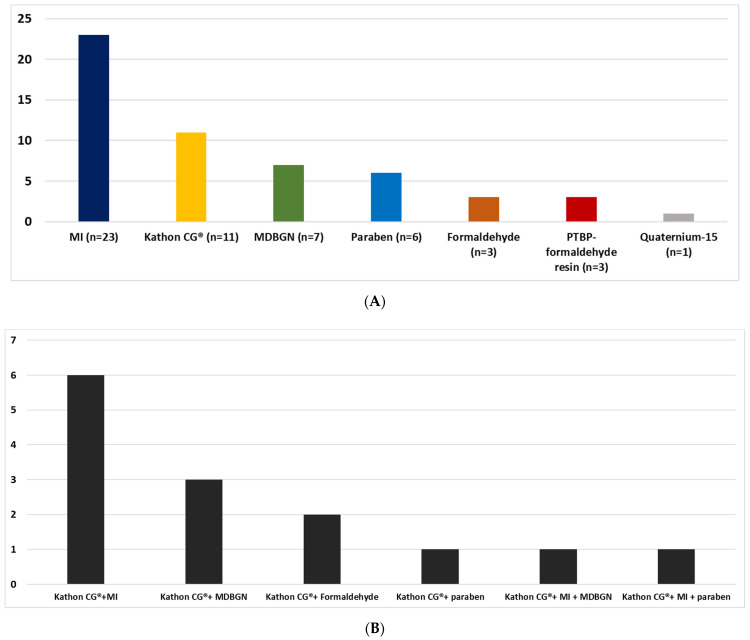
(**A**): Proportion of adult AD patients with one PCHS according to allergens (number of patients). (**B**) Proportion of adult AD patients with two or three PCHSs according to allergen combinations (number of patients).

79.41% of patients had one PCHS (most commons were MI, Kathon CG^®^ and MDBGN), whereas 17.65% had two PCHSs (most common combination: Kathon CG^®^ + MI) and 2.94% had three PCHSs (Kathon CG^®^ + MI + MDBGN/paraben) (Figure 3 and Figure 4A,B).

**Figure 5:**
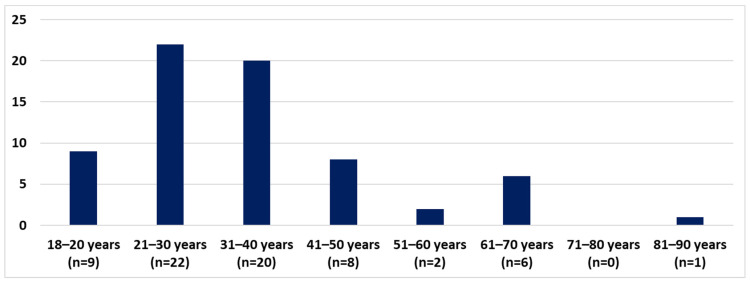
PCHS adult AD patients according to age (number of patients).

**Figure 6:**
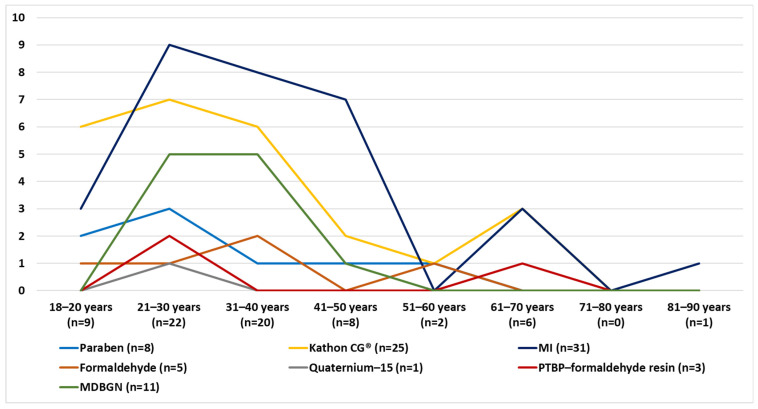
Typical PCHS in adult AD patients according to age groups (number of patients).

3. Age distribution:

PCHS adult AD patients belonged mostly in the 21–30 and 31–40 age groups (Figure 5). According to the age distribution, for CHS caused by preservatives there is a peak in the age group 21–40, with another smaller rise caused by Kathon CG^®^, MI and PTBP formaldehyde resin in the group of 61–70. In the age groups of 51–60 and after 71, PCHS was not typical (Figure 6).

### 3.4. Skin Symptom Localisations According to Preservatives

In most cases of adult AD PCHS patients, the skin symptoms affected the upper and lower limbs. In regard to allergens by paraben, Kathon CG^®^ and MI, the limbs were mostly affected, with a significant neck-face-periorbital region involvement caused by MI. Skin symptoms in the face-neck area are to also be mentioned, caused by MDBGN and Kathon CG^®^. Other regions (mouth, ears, axillae and inguinal region) were mostly affected by MI and MDBGN hypersensitive patients. Widespread skin symptoms were typical in MDBGN, MI and Kathon CG^®^ CHS patients (Table 1).

### 3.5. Concomitant CHS in the PCHS Adult AD Group

The most common concomitant allergens for PCHS adult AD patients were nickel (22 patients (p)), lanolin alcohol (13 p), balsam of Peru (11 p), propylenglycol and thiomersal (10 p), wood tar (9 p), fragrance mix II and thiuram mix (7 p) and mercury-chloride, cobalt and fragrance mix I (6 p) (Table 2).

## 4. Discussion

AD is a chronic inflammatory skin disease which affects about 1–10% of the adults and about 15–20% of the children worldwide [20].

AD has a multifactorial background. Skin barrier dysfunction, immune system dysregulation, the disbalance of the skin bacterial microbiome and genetic factors are also included in the complex pathogenesis. Endogenous and exogenous components can also modify the prognosis of the disease. One of the most remarkable predisposing factors is a family history of atopic diseases. Patients with mutations or impaired expression of the filaggrin gene were also reported, which contribute to the skin barrier. The lipid metabolism with decreased ceramide production is also damaged. Trans-epidermal water loss was increased. All these factors weaken the proper skin barrier functions leading to inflammation of the skin. In this process, initially the type-2 T-helper cells (Th2) (producing mainly IL-4, IL-5, IL-13, IL-25 and IL-31) are crucial, with a subsequent Th2-Th1 cell switch in the chronic phase [1,2,3,4,5,6,7,8,21,22,23].

Not only are the background and the provoking factors of AD rather complex, but also the clinical characteristic shows a dynamically changing tendency over time. In the past, it was believed that AD begins always at a young age (infancy or childhood) and then the skin symptoms disappear with time. According to the recent concepts, AD is considered to be a life-long condition even if the patient has no actual active, inflamed skin lesions at all. It is reported that about 60–80% of the AD population has a very early-onset type of AD. Among them, it is estimated that about 60% of patients have a complete remission before two years of age. The other group of these patients and those who develop AD between 2 and 6 years of age have a higher risk to have chronic and persistent AD. Data of adolescent-onset AD are limited. Most adult AD patients have flare ups after a long symptom-free period or have had persistent AD since childhood. About 2% of AD patients have real adult-onset AD, and are mostly women. The number of elderly patients with active inflammatory AD symptoms is low, although dry and sensitive skin will stay lifelong. A real elderly-onset type of AD is very unusual but possible [1,5,6].

Although the initial onset of the disease can be different among AD patients, the clinical picture of AD is well characterised according to the actual age of the patients. Regarding the connection between age and skin symptom features, infantile, childhood, adolescent/adult and elderly AD types can be defined. The first lesions of infantile AD usually appear some months after birth and are characterised by the acute, exudative skin lesions. AD most commonly affects the scalp and facial regions of the newborns and oozing and crusting are quite typical. The extensor surfaces of the limbs are also predilectional parts for AD in the infants. The childhood AD has a clinical feature of acute and chronic lesions as well. Xerosis and lichenification may also appear. Eczematous skin symptoms usually occur in the bends and in the perioral and periorbital regions. Involvement of the hands and the wrists should also be mentioned. Adolescents and adults have AD skin symptoms often on the head-neck region and the flexural localisations and hands are the most typical places for the clinical distribution of the skin symptoms. Cosmetics and inadequate skin care may exacerbate the skin condition. Hand eczema is a leading problem for adult AD patients and is a great burden on the quality of life. Irritants and environmental contact allergens are typical provoking factors of it. After 60 years of age, widespread AD skin symptoms are uncommon but possible, and even erythroderma may occur. Certain other dermatologic diseases need to be excluded in order to diagnose AD properly in adults (lymphomas, allergic contact dermatitis and drug reactions) [1,5,6].

Having vulnerable skin to the endogenous factors mentioned before, certain exogenous factors may trigger an exacerbation of AD. Among environmental exposure irritants, aero/food/contact allergens, stress, certain microbes (*Staphylococcus aureus*, *Malassezia* and *Trichophyton species*) and pollutants can be mentioned. Studies on the adult AD population attribute a significant role to different kinds of contact and aeroallergens as important provoking factors underlying a sudden flare up of the symptoms or therapy resistance [1,2,3,4,5,6,7,8,21].

Allergic contact dermatitis (ACD) is a cell-mediated, delayed, type IV hypersensitivity reaction. During the sensitization phase, the person comes into contact with the allergen for the first time. Allergens are low-molecular-weight substances connected to a larger carrier (haptens). Haptens are engulfed by antigen-presenting cells migrating to the local lymph nodes where the activation and proliferation of naive T-cells begins. The formerly naive T-cells become allergen-specific T-cells which have a key role during the elicitation phase, since re-exposure of the allergen activates them, provoking inflammation and the clinical picture of ACD. Prevalence of CHS is reported to be up to 20%, and the incidence of ACD seems to be rising. In Europe, 27% of the general population has at least one CHS [11,20,24,25].

Regarding human data, compared to control skin, the absorption through AD skin is increased not only in case of lesional, but non-lesional skin as well. However, there are regional differences in the increased skin absorption. Forehead and genital skin is reported to be more vulnerable than forearm skin, for example. The severity of the AD and the presence of the filaggrin mutation are also factors which should be kept in mind when discussing skin absorption. The more severe and widespread the AD is, the more increased the skin absorption will be. The filaggrin mutation contributes to the higher risk of having a skin barrier impairment in non-lesional skin, too. In conclusion, according to the literature, patients with AD have nearly a twofold-increase in skin absorption of different chemicals, including irritants and contact allergens as well. The most common contact allergens in AD are metals, fragrances, emollients, vehicles, dyes, antibiotics, topical antiseptics and preservatives [2,4,6,7,11,20,26,27,28,29,30].

The number of allergic contact reactions to different cosmetic products is reported to be increasing. The preservatives are the most common cosmetic contact allergens after fragrances, but emulsifiers, vehicle components, sunscreen agents and nail resins can also provoke CH. In the general European population, 6.2% have PCHS. This fact highlights the importance of these allergens, since AD patients regularly use numerous personal care products besides topical medications. These products may contain contact allergens (fragrances, preservatives, vehicle components and emulsifiers) [4,8,20,25,30].

Cosmetics, hygiene products and local therapeutics with high water content need chemical preservation. Seven of the most common preservatives are a part of the EEBS: paraben, Kathon CG^®^, MI, formaldehyde, Quaternium-15, PTBP-formaldehyde-resin and MDBGN [9,12,13,16,19,30].

Parabens are one of the most commonly used preservatives around the world. Contact allergy is reported to parabens from 1940. Nearly 35 variants of parabens are known, but methyl-, ethyl-, propyl-, and butylparabens became the most widely applicated ones. Parabens have an antimicrobial spectrum covering gram-positive bacteria and fungi. The different variants are often combined with each other or with other preservatives as well. Foods, medications and cosmetics also contain this substance. Paraben is a rather common cosmetic ingredient in skin care products (moisturisers, shampoos and hair conditioners) and can also be found in makeup products, powders, foundations, eye contour pencils, mascaras, lipsticks, lip glosses, hair dyes, nail cosmetics, toothpastes and mouthwashes [30,31].

Kathon CG^®^ is the 3:1 mixture of methylchloroisothiazolinone (MCI) and methylisothiazolinone (MI). It has become a quite popular preservative because of its potent antimicrobial effects which covers gram-positive bacteria, gram-negative bacteria, yeasts and moulds. Despite its advantages as a broad-spectrum antimicrobial agent, its contact sensibilisation-provoking effect was also published. Kathon CG^®^ was introduced in the early 1980s as an industrial and household product and cosmetic preservative. However, an increasing tendency in cases of CHS rates was reported, and the first cosmetic-related Kathon CG^®^ contact dermatitis was reported in 1985. MCI and MI were also published to be provoking factors of allergic contact dermatitis in humans, and animal studies also showed that mostly MCI was the main sensitiser out of the mixture. A large variety of cosmetic formulations contain this chemical. It is common in intimate hygiene cosmetics, hair care products and facial cleansers. Shower gels, shampoos, makeup products, moisturisers, body lotions, creams and hair cosmetics are also sources of Kathon CG^®^ exposure [13,16,30,32].

MI was previously used for preservation only as a component of Kathon CG^®^. It was believed to be a less potent sensitising contact allergen than MCI. In the 2000s it was allowed to be an individual preservative; firstly, in industrial products, and later in household products and cosmetics. MI CHS was reported increasingly, and occupational cases and cosmetic-related studies were also published. In cases of occupational sources of exposures, cutting oils, glues, inks, paints and lacquers are remarkable. Among household products, glass cleaners, wood cleaners, laundry detergents, dishwashing liquids and fabric softeners are common products containing MI. MI is an important preservative in personal care products (oils, body lotions and creams), hair cosmetics (shampoos, hair straighteners, hair sprays, conditioners and hair dyes), soaps, deodorants, make-up products (powders, eye contour pencils and eyeshadows) nail cosmetics, aftershaves, moisturisers, self-tanning products, sunscreens and intimate hygiene products [13,14,16,30,33].

Formaldehyde is a gas, which is called formalin when it is an aqueous solution. It has a biocide, preservative and denaturant function in cosmetics. The aqueous formaldehyde solutions are known irritants and their CHS provoking effect has already been published in occupational and non-occupational cases as well. Cosmetic exposures of formaldehyde include shampoos, hair conditioners, hair dye products, soaps, detergents, bath oils, bath salts, personal care products, shaving creams, moisturisers, face masks, face wraps and nail cosmetics. In Europe, different kinds of regulations are present regarding the concentrations of formaldehyde. It is permitted for usage as a preservative at a concentration of 0.2% in cosmetics and 0.1% in oral hygiene products, and products must be labelled as “contains formaldehyde” if they contain more than 0.05% of this chemical [30,34,35].

Quaternium-15 is a formaldehyde-releaser preservative, which was first introduced to be a part of the EEBS in 1984. It is a potent antimicrobial agent, which is effective even at low concentrations. The sources of Quaternium-15 exposure are quite variable, since non-cosmetic and cosmetic products can contain it as well. As non-cosmetic products, detergents, polishes, inks, paints, textile finishing products, joint cements and metalworking fluids can be mentioned. Cosmetic sources of exposure are baby shampoos, body lotions, soaps, detergents, bath salts, eyeliners, eyeshadows, eyeshadow removers, perfumes, hair conditioners, shampoos, hair sprays, hair dyes, face powders, lipsticks, primers, nail cosmetics, deodorants, face creams, body lotions, face wraps and self-tanners [30,36,37].

The PTBP-formaldehyde resin is also a formaldehyde-releaser preservative. Contact allergies provoked by this chemical have been known for decades. The first case of contact dermatitis to PTBP-formaldehyde resin, published in the late 1950s, was caused by a shoe glue. Non-occupational and occupational sources of exposures can be mentioned. Occupational CHS is reported to be less frequent. Workers in the car industry and shoe manufacturing are affected. Most cases of CHS to PBTP-formaldehyde resin are non-occupational. Mainly domestic glues, amputation prostheses, leather watch straps and neoprene orthopaedic knee braces belong to this group. However, PTBP-formaldehyde resin as a preservative can also be found in nail cosmetics and deodorants [38,39].

Methyldibromo-glutaronitrile is a preservative and known contact allergen with mainly cosmetic sources of exposure. Although MDBGN was banned in Europe, not only from leave-on products in 2005 but also from rinse-off products, CHS reported about this preservative is still present nowadays. Among cosmetics, MDBGN is used for preservation in shampoos, soaps, cleansers, body lotions, make-up products and make-up-removing wet wipes. [19,40,41].

Due to the long-term usage of a large number of topical preparations (cosmetics and medications), AD patients are reported to be more likely to develop CHS not only to fragrances, but to preservatives as well. However, this topic is not researched in more detail and data on PCHS in adult AD patients are very limited [8,9,10,11].

In our 15-year (2007–2021) retrospective study we examined the clinical features of PCHS in adult AD patients. The rate of adult PCHS AD patients (9.4%) is remarkable in the total PCHS population, in our overall tested adult AD population (10.6%) and in the AD population with at least one CHS (17.4%) as well.

According to our observation in adult AD patients the most common preservatives are MI, Kathon CG^®^ and MDBGN despite the fact that MI was patch tested only from 2014. By concomitant PCHS the most common combination was Kathon CG^®^ + MI.

The most affected adult PCHS AD patients belonged to the age group of 21–30 and most skin symptoms were localised to the limbs and face-neck region.

According to our data, besides metals, the most common other EEBS concomitant allergens were cosmetic-therapeutic ones (lanolin alcohol, balsam of Peru, propylenglycol, wood tar and fragrance mix I and II) in the PCHS adult AD group.

To our best knowledge, this is the first study which focuses on the clinical characteristics of PCHS in the adult AD group.

In conclusion, PCHS is important among adult AD patients. This finding highlights that adult AD patients are worth patch testing in case of therapy-resistance or worsening skin symptoms due to topical medications and/or personal care products. Our results underline the importance of regular and detailed medical counselling about conscious skin care and about applying not only fragrance-free, but also preservative-free products in this population.

## Figures and Tables

**Table 1:** Localisation of skin symptoms according to preservatives (number of patients).

	Paraben	Kathon CG^®^	MI	Formaldehyde	Quaternium-15	PTBP-Formaldehyde Resin	MDBGN
**Face**	3	**6**	**12**	2	0	2	**8**
**Periorbital region**	0	**5**	**11**	3	1	2	**6**
**Scalp**	0	3	6	2	0	0	6
**Neck**	1	4	**8**	3	0	0	**7**
**Upper limbs**	**7**	**21**	**23**	4	0	1	**11**
**Lower limbs**	**4**	**10**	**14**	3	0	0	**7**
**Trunk**	1	5	10	2	0	0	6
**Anogenital-gluteal region**	1	4	5	2	0	0	6
**Other (mouth, ears, axillae, inguinal region)**	3	5	**11**	2	0	1	**7**

**Table 2:** Concomitant CHS of PCHS adult AD patients in the EEBS.

Allergen (cc)	PCHS Adult AD Patients (*n* = 68)	Period
Nickel (II)-sulphate hexahydrate (5%)	**22**	2007-
Lanolin alcohol (30%)	**13**	2007-
Balsam of Peru (25%)	**11**	2007-
Propylenglycol (20%)	**10**	2007-
Thiomersal (0.1%)	**10**	2007-
Wood tar (12%)	**9**	2007-
Fragrance mix II (14%)	**7**	2007-
Thiuram mix (1%)	**7**	2007-
Mercury-chloride (0.1%)	**6**	2007-
Cobalt (II)-chloride hexahydrate (1%)	**6**	2007-
Fragrance mix I (8%)	**6**	2007-
Mercury (II)-amidochloride (1%)	5	2007-
Potassium dichromate (0,5%)	5	2007-
PPD (4-phenylendiamine base) (1%)	5	2007-
Propolis (10%)	5	2007-
Budesonide (0.1%)	3	2007-
Colophony (20%)	3	2007-
Lyral^®^ (Hydroxyisohexyl 3-cyclohexene carboxaldehyde) (5%)	3	2018-
Evernia furfuracea (tree moss) (1%)	3	19 February 2018
Benzocaine (5%)	2	2007
Iodchlore-oxychinoline (clioquinol) (5%)	1	2007
Tixocortol-21-pivalate (1%)	1	2007
Primin (0.01%)	1	2007
N-isopropyl-N′-phenyl-p-phenylenediamine (IPPD) (0.1%)	1	2007
Resorcin (2%)	1	2007
2-Mercaptobenzothiazole (MBT) (2%)	1	2007
Cocamidopropyl betaine (1%)	1	2013
Decyl-glycoside (5%)	1	2018
Methyl-methacrylate (2%)	1	2017- (98 patients in 2016)
Bisphenol A (epoxy resin) (1%)	0	2007-
Lavender oil (2%)	0	2013-
2-hydroxyethyl-methacrylate (2%)	0	2017- (98 patients in 2016)
Ethyl-acrylate (0.1%)	0	2017- (98 patients in 2016)
d-Limonene (10%)	0	24 October 2017-
Linalool (10%)	0	24 October 2017-
Lauryl-glycoside (3%)	0	2018-
Sorbitan sesquioleate (20%)	0	16 August 2019-
Turpentine oil (0.3%)	0	2007–2017
Sesquiterpene lactone (0.1%)	0	2007-
Phenylbutazone (10%)	0	2007-

Paraben mix (16%), Kathon CG^®^ (methylchloroisothiazolinone/methylisothiazolinone [MCI/MI) 3:1) (0.01%), methylisothiazolinone (0,2%), formaldehyde (2%), Quaternium-15 (Dowicil 200) (1%), para-tert-butylphenol-formaldehyde-resin (1%), methyldibromo-glutaronitrile (MDBGN) (0.3%).
